# Triple Design Strategy for Quinoxaline-Based Hole Transport Materials in Flexible Perovskite Solar Cells

**DOI:** 10.3390/molecules30051129

**Published:** 2025-02-28

**Authors:** Yuanqiong Lin, Zeyuan Gao, Xiaoshang Zhong, Yinghua Lu, Song Tu, Xin Li

**Affiliations:** 1 Pen-Tung Sah Institute of Micro-Nano Science and Technology, Xiamen University, Xiamen 361005, China; yuanqionglin@stu.xmu.edu.cn; 2 School of Electronic Science and Engineering, Xiamen University, Xiamen 361005, China; 36120231150453@stu.xmu.edu.cn; 3 College of Chemistry and Chemical Engineering, Xiamen University, Xiamen 361005, China; 36520231152088@stu.xmu.edu.cn (X.Z.); ylu@xmu.edu.cn (Y.L.)

**Keywords:** hole transport material, noncovalent conformational locks, self-assembly, donor–acceptor strategy, perovskite solar cells

## Abstract

Molecular design strategies such as noncovalent conformational locks, self-assembly, and D-A molecular skeletons have been extensively used to devise efficient and stable hole transport materials. Nevertheless, most of the existing excellent examples involve only single or dual strategies, and triple strategies remain scarcely reported. Herein, we attempt to develop two quinoxaline-based hole transport materials (**DQC-T** and **DQ-T-QD**) through a triple strategy encompassing an S···N noncovalent conformational lock, D-A molecular skeletons, and self-assembly or conjugate engineering. The S···N noncovalent conformational lock formed by thiophene sulfur atoms and quinoxaline nitrogen atoms improves molecular planarity, further inducing the formation of high-quality perovskite films and enhancing hole transport ability; the asymmetric D-A molecular backbone endows the material with a larger dipole moment (*μ* = 5.80 D) to promote intramolecular charge transfer; and the carboxyl group, methoxy, and sulfur atom establish strong interactions between the NiO_x_ and perovskite layers, including self-assembly and defect passivation, which mitigates the occurrence of detrimental interfacial charge recombination and reactions. Thus, the 2-thiophenecarboxylic acid derivative **DQC-T**, featuring an asymmetric D-A molecular backbone, exhibits superiority in terms of good interface contact, hole extraction, and transport compared to **DQ-T-QD** with a D-A-π-A-D type structure. Naturally, the optimal power conversion efficiency of NiO_x_/**DQC-T**-based p-i-n flexible perovskite solar cells is 18.12%, surpassing that of NiO_x_/**DQ-T-QD**-based devices (16.67%) and NiO_x_-based devices with or without **DQC** (a benzoic acid derivative without a noncovalent conformational lock) as co-HTMs (16.75% or 15.52%). Our results reflect the structure–performance relationship well, and provide a referable triple strategy for the design of new hole transport materials.

## 1. Introduction

Inverted (p-i-n) perovskite solar cells (PSCs) are deemed to be photovoltaic devices with broad development prospects because of their outstanding stability, simple device preparation process, and excellent compatibility with flexible substrates [1,2,3]. Currently, poly[bis(4-phenyl)(2,4,6-trimethylphenyl)amine (PTAA), self-assembled molecules (SAMs), and NiO_x_ are prevalent hole transport materials (HTMs) for p-i-n PSCs. However, their respective limitations should not be overlooked, such as the large batch variation of PTAA [4,5], poor wettability of SAMs [6,7,8], and high defect density of NiO_x_ [9,10]. Therefore, it is important to develop new efficient and stable HTMs as alternatives to commonly used HTMs or as co-HTMs.

In view of this, diverse molecular design strategies have been adopted to tailor a large number of structurally diverse hole transport materials. Yang and his cooperative partner utilized a noncovalent conformational lock strategy to design and synthesize a couple of bithiophene-based HTMs featuring an S···O noncovalent conformational lock, codenamed BTORA and BTORCNA [11]. BTORA and BTORCNA integrated the merits of excellent thermostability, uniform film surface, and enhanced film crystallinity. In comparison, BTORCNA with a cyano group exhibited a more efficient defect passivation and deeper energy levels. As a result, BTORCNA-based p-i-n PSCs created a champion PCE of 21.1%. Wang et al. developed pyrrolo [3,2-*b*]pyrrole-based HTMs (6FPPY) based on a multi-fluoridation strategy [12]. The incorporation of 6FPPY into p-i-n PSCs efficiently solved the undesired interfacial issues between NiO_x_/perovskite, including high defect density, deleterious interfacial reactions, and interface energy level mismatch, thus enhancing hole transport capability. The PCE of the device prepared with 6FPPY was as high as 24.0%, outperforming that of a NiO_x_-based device. Recently, Zhou et al. proposed the introduction of glycol monomethyl ether side chains (GMs) into the [4-(9H-Carbazol-9-yl)butyl]phosphonic acid (4PACz) structure to augment the wettability of perovskite precursors on the hole transport layer [13]. Additionally, the GM group played an active role in defect passivation and energy level regulation. Ultimately, the resulting HTM (GM-4PACz) was successfully applied in stable p-i-n PSCs, attaining an impressive PCE of 25.5%. However, most reported HTMs were tailored through single or dual strategies, rendering it arduous to balance multiple properties simultaneously. The poor solubility and film quality resulting from a large conjugate system, the reduced hole mobility and conductivity due to flexible alkyl or alkoxy chains, and the large molecular volume and shallower HOMO energy levels as a consequence of the traditional noncovalent conformational lock all corroborated the above points [14,15,16,17]. Additionally, there is a large research gap in the triple molecular design strategy for HTMs.

In this work, we constructed asymmetric D-A-type HTMs (**DQC-T**) and symmetric D-A-π-A-D-type HTMs (**DQ-T-QD**) by employing a triple design strategy of noncovalent conformational locking, D-A organic molecular backbone, and self-assembly or conjugate engineering. Subsequently, the focus was on revealing the effects of noncovalent conformational locking and structural symmetry on material properties and device performance. The D-A molecular backbone consisted of dibenzofuran-substituted diphenylamine (donor) with a strong electron-donating capacity and quinoxaline (acceptor) with a moderate electron-withdrawing capacity [18,19,20,21]; the π-bridge was a thiophene ring, where a sulfur atom formed a robust S···N noncovalent conformation lock with a nitrogen atom on the adjacent quinoxaline unit [22,23]; and the anchoring group involved was a carboxyl group, which underwent a self-assembly on the NiO_x_ layer. The results showed that the presence of S···N noncovalent conformation locks should well balance the film-forming properties and hole mobility of materials, as evidenced by the significant reduction in the dihedral angle between the associated units (e.g., quinoxaline and neighboring aromatic rings) and the formation of a high-quality film morphology. A well-matched asymmetric D-A molecular backbone should be conducive to increasing the molecular dipole moment, thereby improving the intramolecular charge transfer capability. Additionally, the acid anchoring group (e.g., carboxyl group) and electron-rich functional groups (e.g., thiophene and methoxy) should be regarded as synergetic sites that inhibited unacceptable reactions or charge recombinations at the NiO_x_/perovskite interface, enabling more efficient hole extraction and transport. Consequently, **DQC-T** and **DQ-T-QD** were utilized in p-i-n flexible PSCs with the advantages of light weight, solution processability, and flexible bendability [24,25,26]. Additionally, the device with **DQC-T** and NiO_x_ as co-HTMs yielded a higher PCE of 18.12%. Our work validates that a triple design strategy can endow HTMs with superior properties, leading to improved device performance.

## 2. Results and Discussion

**Figure 1:**
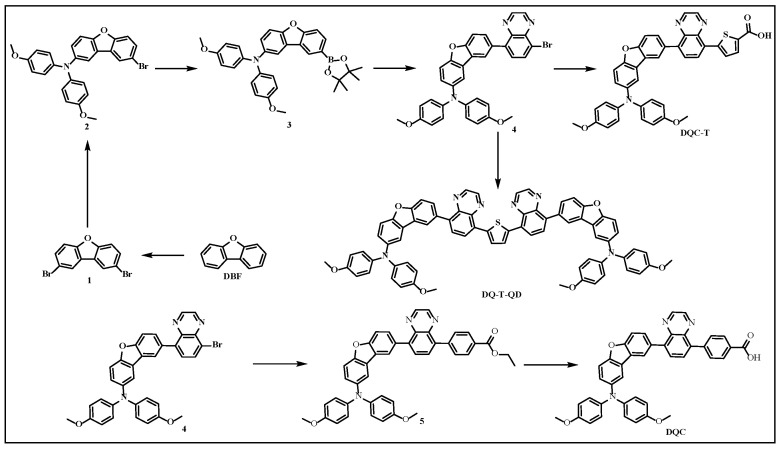
The synthetic routes of two quinoxaline-based HTMs.

A tripe design strategy containing an S···N noncovalent conformation lock, D-A organic molecular skeleton, and self-assembly or conjugation engineering was employed in the design of asymmetric D-A-type HTMs (**DQC-T**) with a carboxylic acid anchoring group and symmetric D-A-π-A-D-type HTMs (**DQ-T-QD**). This work highlighted the positive effects of combining multiple design strategies to improve material properties and device performance, and disclosed structure–property relationships. As illustrated in Figure 1, the construction of C-N and C-C bonds in the target compound structure required the participation of common Buchwald–Hartwig coupling, Suzuki coupling, and Miyaura borylation reactions. Additionally, the structures of all compounds were identified by ^1^H NMR and ^13^C NMR (Figures S1–S4 in Supplementary Materials).

**Figure 2:**
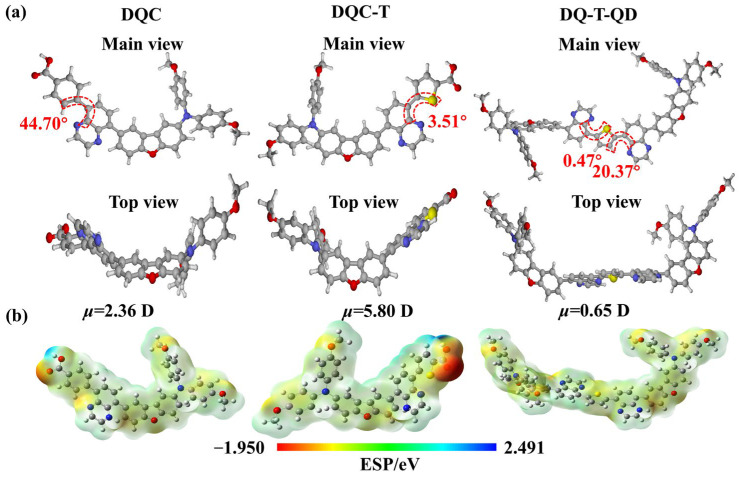
(**a**) The optimized geometries; (**b**) ESPs of **DQC**, **DQC-T**, and **DQ-T-QD** based on DFT.

The optimal conformation and energy level electron cloud distribution of HTMs were investigated by DFT calculation. The optimized geometric structure (Figure 2a) revealed that the rigid planes of the dibenzofuran, quinoxaline, and thiophene fragments helped to enhance molecular planarity, which was expected to lead to better π–π stacking of the molecules. Unlike in **DQC**, the π-bridge in the **DQC-T** structure was a thiophene ring rather than a benzene ring, which significantly reduced the dihedral angle between the quinoxaline and the adjacent aromatic ring to 3.51°. As for **DQ-T-QD**, it possessed a symmetric structural unit and larger π-conjugation system, and its optimal conformation exhibited approximate central symmetry. Its thiophene ring could form different chemical bonds with the quinoxaline units on either side, including an S···N noncovalent conformational lock and N···H bond, resulting in smaller dihedral angles of 0.47° and 20.37°, respectively. The apparent increase in molecular planarity of **DQC-T** and **DQ-T-QD** was ascribed to the smaller molecular size of thiophene and the construction of S···N noncovalent conformational locks in the molecular structure. The flexible noncovalent conformational locks improved the planarity of the molecule while ensuring the film-forming properties of the material. It could be further hypothesized that the introduction of non-covalent conformational locks in the molecular structure facilitates an increase in the hole transport capacity of the material. In addition, according to the frontier molecular orbital model simulated by DFT (Figure S5), the HOMO electron density of **DQC-T** and **DQ-T-QD** was mainly distributed in the triphenylamine portion constructed from the benzene ring unit of dibenzofuran and bis(4-methoxyphenyl)amine, whereas the LUMO electron density was shifted to the electron-deficient quinoxaline unit or even to thiophene. This HOMO–LUMO positional gap favored intramolecular carrier transport. The corresponding HOMO and LUMO energy levels were further obtained from DFT calculations, as shown in Table 1. For the two HTMs designed in this work, the symmetry of the structure had less influence on the energy levels.

The dipole moments of **DQC-T** and **DQ-T-QD** were also calculated (Figure 2b) to evaluate the intramolecular charge transport capacity. Obviously, the molecular dipole moment of **DQC-T** (5.80 D) was much larger than that of **DQ-T-QD** (0.65 D) and **DQC** (2.36 D), which arose from the synergistic effect of the asymmetric D-A structure and the incorporation of thiophene and a carboxyl group [17,27]. It could be concluded that **DQC-T** possessed an extremely strong intramolecular charge transfer ability. The electrostatic potential (ESP) surfaces of HTMs were studied to further visualize the electron density distribution of the molecules and to understand the sites where the materials interacted with perovskite and NiO_x_. As depicted in Figure 2b, the positive potentials of the three HTMs were mainly dispersed on the dibenzofuran-substituted diphenylamine and quinoxaline units, while the negative potentials were mainly concentrated on the methoxy group, thiophene, and carboxyl group. On the one hand, the negative potential on the S atom of thiophene was evidence of the existence of an S···N noncovalent conformational lock in the molecular structure. On the other hand, all of the electron-rich functional groups mentioned above could act as Lewis bases to form coordination bonds with uncoordinated Pb^2+^, thereby passivating the perovskite defects. Even carboxylic acids could undergo strong self-assembly on the NiO_x_ layer. Among them, the carboxyl group behaved with the most pronounced negative potentials. It can also be surmised that the carboxyl group has the strongest defect passivation and self-assembly effects.

**Figure 3:**
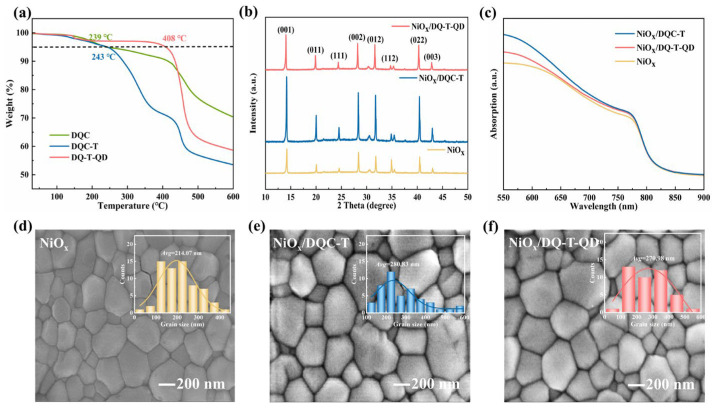
(**a**) TGA curves; (**b**) XRD patterns; (**c**) UV–vis absorption spectra; (**d**–**f**) SEM images (magnification: 50 k) of perovskite films deposited on different substrates.

Considering the importance of the thermal properties of HTMs to the stable operation of the device, the TGA and DSC curves of **DQC-T** and **DQ-T-QD** were analyzed (Figure 3a and Figure S6). It was found that the decomposition temperatures (*T*_d_) of **DQC**, **DQC-T,** and **DQ-T-QD** were about 239, 243, and 408 °C, respectively. The high *T*_d_ avoided the risk of their decomposition during p-i-n fPSC fabrication and operation. Notably, **DQ-T-QD,** without a carboxylic acid group, possessed a higher *T*_d_ due to its larger volume and molecular weight, which was conducive to the formation of a more stable hole transport layer film [28,29]. In the tested DSC curves, no glass transition temperature (*T*_g_) was observed for both **DQC-T** and **DQ-T-QD**, whereas the latter revealed a clear melting heat absorption peak with a melting temperature (*T*_m_) of 153 °C. Furthermore, both demonstrated characteristic exothermic transitions at approximately 242 °C in their DSC profiles. It was noteworthy that **DQC-T** tended to undergo thermal decomposition at this temperature range, where **DQ-T-QD** might have experienced a recrystallization process [30], as evidenced by their markedly different thermal stabilities observed in the TGA analysis. To sum up, the designed and synthesized HTMs had sufficient thermal stability and met the requirements of device preparation.

Both the normalized and raw UV–visible absorption spectra of **DQC-T** and **DQ-T-QD** in dichloromethane solution are exhibited in Figure S7, and the detailed photophysical parameters are summarized in Table 1. The strongest absorption peaks of **DQC-T** and **DQ-T-QD** appeared at 303 and 304 nm, respectively, which belonged to the π–π* transition of the corresponding molecular conjugation system. Notably, compared with **DQC-T**, **DQ-T-QD** exhibited a slight red-shift in its maximum absorption wavelength, accompanied by a distinct increase in absorbance. This phenomenon could be ascribed to the extended conjugate system in **DQ-T-QD** [31,32]. For **DQC-T**, another weaker absorption peak at 385 nm confirmed the intramolecular charge transfer process between its dibenzofuran-substituted diphenylamine and the quinoxaline unit. As for **DQ-T-QD**, its shoulder peak near 371 nm suggested that the longer π-conjugated chain brought about by its D-A–π–A-D molecular backbone further induced the stronger interchain interactions [33]. Additionally, for both, the absence of strong absorption peaks in the region of 400–760 nm was good news for light absorption in the perovskite active layer. Meanwhile, **DQC-T** and **DQ-T-QD** were subjected to cyclic voltammetry tests to assess the influence of involved structural adjustments on the energy levels of the materials. As shown in Figure S8, these two HTMs have similarly shaped and highly reversible redox peak pairs, indicating their excellent electrochemical stability. Based on the onset oxidation potentials, the corresponding HOMO energy levels were calculated to be −5.29 and −5.31 eV, respectively. Combined with the HOMO energy and the optical band gaps derived from the UV–vis edge, the LUMO energy levels were further reckoned to be −2.52 and −2.33 eV, respectively. The variance in LUMO energy level might arise from differences in the extent of molecular aggregation, as well as disparities in the effective lengths of the additional conjugation units involved [32]. It could be seen that the energy levels of **DQC-T** and **DQ-T-QD** met the energy level requirements for hole transport materials. This conclusion was consistent with that of the obtained DFT calculations. Additionally, in the fluorescence emission spectra, **DQC-T** and **DQ-T-QD** displayed clear emission peaks at 488 and 462 nm, respectively (Figure S9). HTMs, with their excellent fluorescence emission properties, convert UV light into visible light that can be absorbed by perovskite, thereby increasing the utilization of solar irradiation [34]. The corresponding Stokes shifts were also calculated from the emission peaks, which were 185 and 152 nm, respectively. Compared with **DQ-T-QD**, the structure of **DQC-T,** featuring a larger Stoker shift, would change greatly in the excited state.

To investigate the influence of the HTMs’ molecular structure on the properties of perovskite thin films, XRD patterns, UV–vis spectra, and SEM images were recorded (Figure 3). The XRD pattern first revealed the crystallization of perovskite films deposited on substrates with or without HTM modification. The involved perovskite films showed a distinct set of diffraction peaks corresponding to the typical α-phase of Cs_0.05_(FA_0.92_MA_0.08_)_0.95_Pb(I_0.92_Br_0.08_)_3_ film. Comparing the intensity of the diffraction peaks, it could be speculated that the introduction of **DQC-T** and **DQ-T-QD** was favorable for the generation of larger size perovskite grains, as illustrated by the UV–vis absorption spectra (Figure 3c). Meanwhile, with HTM modification, the visible light absorption of the perovskite films was indeed significantly enhanced, which supplemented the results of the fluorescence emission spectra. SEM measurements (Figure 3d–f and Figure S10) were used to further visually characterize the morphology of perovskite films. It was observed that the perovskite films covered on a bare NiO_x_ layer had more small size grains and cracks. The morphology of the perovskite films was optimized to a greater extent after HTM modification. Notably, the perovskite film deposited on the NiO_x_/**DQC-T** modified substrate had the largest average grain size (≈280.83 nm) and no significant holes and cracks were observed. This demonstrated the utilization of **DQC-T**, which incorporated both a carboxyl group and an asymmetric D-A backbone, enabled optimal interfacial contact at the NiO_x_/perovskite interface, and consequently facilitated the formation of perovskite crystals with larger grain sizes.

**Figure 4:**
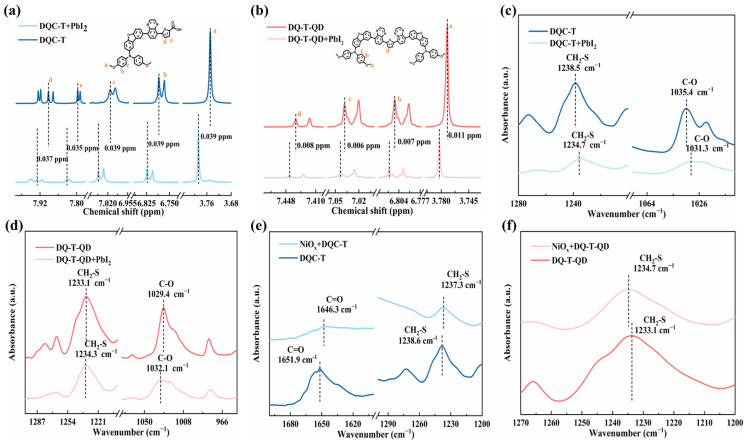
(**a**,**b**) The ^1^H NMR spectra and (**c**,**d**) ATR-FTIR spectra of different HTMs and HTMs/PbI_2_; (**e**,**f**) ATR-FTIR spectra of different HTMs and HTMs/NiO_x_.

In order to ulteriorly explore the interaction between the HTMs and perovskite, the ^1^H NMR spectra and ATR-FTIR spectra of the pure HTM samples and HTM/PbI_2_ mixture samples were analyzed and compared (Figure 4). Compared to the pure **DQC-T** sample, the ^1^H NMR spectra measured for the **DQC-T**/PbI_2_ mixture showed significant shifts of both hydrogen atoms on the methoxydianiline unit (H_a_, H_b_, and H_c_) and the thiophene unit (H_d_ and H_e_) towards the lower field. Additionally, the offset values for specific chemical shifts ranged from 0.035 to 0.039 ppm, which was favorable evidence for the presence of strong C-O···Pb and S···Pb bonds. The occurrence of the above interactions reduced the electron cloud densities of the oxygen and sulfur atoms, which in turn triggered the chemical shifts of the H atoms in the neighbouring aromatic rings to move to the lower field [35,36]. When **DQ-T-QD** was mixed with PbI_2_, the slight chemical shifts (ranging from 0.006 to 0.011 ppm) of the associated hydrogen atoms suggested that only weak C-O···Pb and S···Pb bonds were formed between **DQ-T-QD** and the perovskite layer. Moreover, in the ATR-FTIR spectra, upon blending with PbI_2_, the CH_2_-S peak on the thiophene unit and C-O peak on methoxy in the structure of **DQC-T** and **DQ-T-QD** were observed to be shifted. This phenomenon again declared the coordination interaction between HTMs and uncoordinated Pb^2+^ in the perovskite layer. In contrast, **DQC-T** could passivate perovskite defects more efficiently, which was a good explanation for the formation of better morphology of perovskite films induced by **DQC-T**.

Meanwhile, the interaction between the involved HTMs and NiO_x_ layers was briefly investigated using ATR-FTIR measurements, and the results are shown in Figure 4e,f. When **DQC-T** was blended with NiO_x_ powder, its C=O characteristic peak drifted from 1651.9 cm^−1^ to 1646.3 cm^−1^, while the CH_2_-S characteristic peak of the thiophene unit only shifted by 1.3 cm^−1^. This proved that at the **DQC-T**/NiO_x_ interface, the C=O-Ni interaction was strong, while the S···Ni interaction was relatively weak [37]. That is, **DQC-T** possessed a strong propensity for self-assembly on the NiO_x_ layer. As for **DQ-T-QD**, the CH_2_-S characteristic peak of its thiophene unit (1233.1 cm^−1^) was also shifted to 1234.7 cm^−1^ after the addition of NiO_x_ powder, disclosing the presence of a relatively weak S···Ni between **DQ-T-QD** and the NiO_x_ layer. Therefore, the incorporation of both HTMs could play a beneficial role in optimizing the interfacial contacts between NiO_x_ and perovskite.

**Figure 5:**
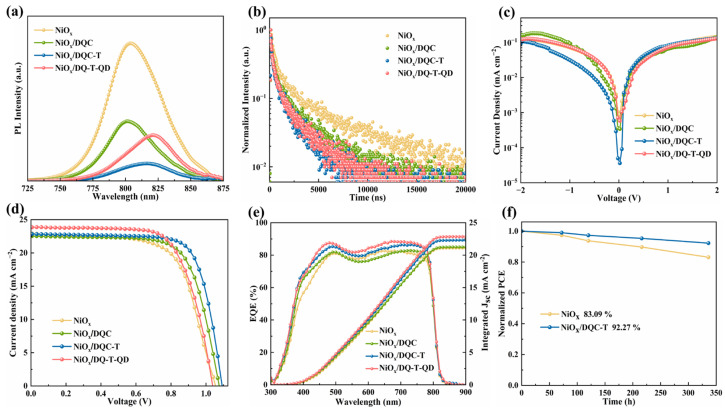
(**a**) PL and (**b**) TRPL spectra of perovskite films deposited on different substrates; (**c**) dark *J–V* curves of different HTMs; (**d**) current density–voltage *(J*–*V*) curves of the PSCs based on different HTMs; (**e**) EQE spectra and integrated *J*_sc_ of the device; (**f**) the normalized stability of NiO_x_**/DQC-T**- and NiO_x_-based unencapsulated flexible devices in an air environment with 30 ± 5% relative humidity.

The steady-state photoluminescence (PL, Figure 5a) and time-resolved photoluminescence spectroscopy (TRPL, Figure 5b) tests were performed to uncover the effect of different HTMs on the hole extraction and transport properties at the interface. After inserting the HTMs between the NiO_x_ and perovskite layers, the PL intensities show a significant quench effect, indicating that **DQC**, **DQC-T,** and **DQ-T-QD** were effective in improving the hole extraction and transport. In contrast, the NiO_x_/**DQC-T**/perovskite film was found to have the highest PL quenching efficiency, which implies it is the fastest hole transfer at the interface. Furthermore, the TRPL curves were fitted with double exponential functions [38] and the average lifetime (*τ*_ave_) of the interfacial charge was derived, with detailed parameters shown in Table S1. The *τ*_ave_ values of the perovskite films on bare NiO_x_ and the NiO_x_/**DQC**, NiO_x_/**DQC-T**, and NiO_x_/**DQ-T-QD** modified substrates were found to be 2503.48, 1614.88, 796.18, and 865.68 ns, respectively. Clearly, the device based on NiO_x_/**DQC-T** could undergo a faster hole extraction process, attributed to **DQC-T**’s moderate molecular planarity and improved interface contact. This was consistent with the conclusions obtained from the PL test analysis. Additionally, the dark current profiles of the relevant devices were obtained to determine the leakage current due to carrier recombination. As shown in Figure 5c, the NiO_x_/**DQC-T**-based flexible devices had lower reverse saturation current density, implying that the introduction of **DQC-T** could well suppress the interfacial non-radiative recombination, which was conducive to obtaining high *V*_oc_ and FF.

To discuss the effect of tuning the molecular structure of the HTMs on their device efficiencies, the current–voltage (*J*–*V*) characteristic curves of the p-i-n fPSCs were collected (Figure 5d and Table 2), where the device structure was PET/ITO/NiO_x_/HTMs/Cs_0.05_(FA_0.92_MA_0.08_)_0.95_Pb(I_0.92_Br_0.08_)_3_/BCP/PCBM/Ag. The device employing bare NiO_x_ as the HTM yielded a lower PCE of 15.52% with a short-circuit current density (*J*_sc_) of 22.36 mA cm^−2^, an open-circuit voltage (*V*_oc_) of 1.05 V, and a fill factor (FF) of 65.96%. The device based on NiO_x_/**DQC** demonstrated a PCE of 16.75%, with a *J*_sc_ of 21.99 mA cm^−2^, *V*_oc_ of 1.08 V, and FF of 70.82%. The NiO_x_/**DQ-T-QD**-based device exhibited a PCE of 16.67% with a *J*_sc_ of 23.25 mA cm^−2^, *V*_oc_ of 1.04 V, and FF of 69.10%. The devices with NiO_x_/**DQC-T** as the bilayer hole transport layer delivered a *J*_sc_ of 22.28 mA cm^−2^, *V*_oc_ of 1.09 V, and FF of 74.44%, achieving a PCE of 18.12%. The modification of the NiO_x_/perovskite interface using asymmetric/symmetric HTMs with an S···N noncovalent conformational lock were all effective in enhancing the cell efficiency. Among them, the NiO_x_/**DQC-T**-based device obtained the champion PCE due to its higher *V*_oc_ and FF values, which stemmed from the reduction of non-radiative recombination. Subsequently, the external quantum efficiency (EQE) spectra of the devices and the corresponding integrated currents were measured, and the results are shown in Figure 5e. All the devices had a high optical response in the range of 300–900 nm, and the difference between the *J*_sc-in_ value obtained from integration and the *J*_sc_ value measured from the *J*–*V* curve was less than 5%. This result further ensured the reliability of the *J*–*V* curve. At the same time, we selected the NiO_x_/**DQC-T**-based device with the best device performance and the NiO_x_-based device as the reference device to evaluate the environmental stability. The tested devices were all unpackaged devices and stored under specific conditions of T = 25 °C and RH = 30 ± 5%. As depicted in Figure 5f, after 336 h of storage, the PCE of the NiO_x_/**DQC-T**-based flexible device was still 92.27% of the initial PCE. The NiO_x_/**DQC-T**-based device exhibited slightly better device environmental stability compared to the NiO_x_-based device, attributed to the fact that the former had better NiO_x_/HTMs/perovskite interfacial contacts.

## 3. Materials and Methods

All the chemical reagents used for compound synthesis and purification were purchased from pharmaceutical suppliers, such as Bidepharm (Shanghai, China), J&K Scientific (Beijing, China), Macklin (Shanghai, China), Energy Chemical (Shanghai, China), Titan (Shanghai, China) and Sinopharm (Beijing, China). They did not need to be reprocessed before use.

A variety of the chemicals and substrates used in the preparation of flexible devices are also commercially available. PET/ITO substrates (14 ohm square^−1^) were provided by Peccell (Tokyo, Japan). Nickel nitrate hexahydrate (Ni(NO_3_)_2_·6H_2_O) (98%) was originated from Sinopharm Chemical Reagent Co. Ltd. (Beijing, China). Formamidinium iodide (FAI) and methylammonium iodide (MAI, 99.8%) were bought from Greatcell Solar Materials Pty Ltd. (Queanbeyan, NSW, Australia). Lead iodide (PbI_2_, 99.99%) and bathocuproine (BCP, 99%) were supplied by Xi’an Polymer Light Co. Ltd. (Xi’an, China). Cesium iodide (CsI, 99.999%) and PCBM (99.9%) came from Advanced Election Technology (Liaoning, China). Additionally, the involved solvents were obtained from Sigma-Aldrich (St. Louis, MO, USA).

### 3.1. Synthesis of ***DQC-T*** and ***DQ-T-QD***

The synthesis details of 2,8-dibromodibenzo[*b*,*d*]furan (**1**) and 8-bromo-*N*,*N*-bis(4-methoxyphenyl)dibenzo[*b*,*d*]furan-2-amine (**2**) directly referred to our published work [39].

Synthesis of *N*,*N*-bis(4-methoxyphenyl)-8-(4,4,5,5-tetramethyl-1,3,2-dioxaborolan-2-yl)dibenzo[*b*,*d*]furan-2-amine (**3**): A 50 mL two-necked round-bottomed flask was charged successively with compound **2** (0.95 g, 2.00 mmol), bis(pinacolato)diboron (B_2_pin_2_, 1.02 g, 4.00 mmol), [1,1′-bis(diphenylphosphino)ferrocene]dichloropalladium(II) (Pd(dppf)Cl_2_, 0.37 g, 0.50 mmol), and CH_3_COOK (0.59 g, 6.00 mmol). Subsequently, the reaction flask was purged with argon gas. Then, commercially available ultra-dry *N*,*N*-dimethylformamide (16 mL) was added via syringe and the mixture was reacted in an oil bath at 80 °C overnight. When the reaction was completed and cooled to room temperature, the resulting mixture was purified by extraction followed by column chromatography to afford compound **3** (milky white solid, 792 mg, 75.9%). ^1^H NMR (500 MHz, CDCl_3_, ppm): δ 1.36 (s, 12H), 3.81 (s, 6H), 6.82~6.84 (m, 4H), 7.03~7.05 (m, 4H), 7.14~7.15 (m, 1H), 7.37~7.39 (m, 1H), 7.50~7.52 (m, 2H), 7.87~7.88 (m, 1H), 8.25 (s, 1H); ^13^C NMR (125 MHz, CDCl_3_, ppm): δ 24.91, 55.54, 83.88, 111.14, 111.85, 114.43, 114.73, 122.88, 124.05, 124.77, 125.54, 127.83, 133.72, 141.99, 144.84, 151.65, 155.34, 159.05.

Synthesis of 8-(8-bromoquinoxalin-5-yl)-*N*,*N*-bis(4-methoxyphenyl)dibenzo[*b*,*d*]furan-2-amine (**4**): The compound 5,8-dibromobenzopyrazine (2.00 g, 6.95 mmol), **3** (1.45 g, 2.78 mmol) and tetrakis(triphenylphosphine)palladium (Pd(PPh)_3_, 0.25 g, 0.22 mmol) were accurately weighed and thoroughly blended within a 250 mL two-necked round-bottomed flask. Then, anhydrous ethanol (16 mL), toluene (70 mL), and K_2_CO_3_ aqueous solution (2M, 16 mL), which had undergone prior deoxygenation treatment, were added to the flask containing the reagents essential for the reaction. Immediately thereafter, the reaction mixture was transferred to an oil bath maintained at 100 °C and allowed to react under an argon atmosphere. After 18 h of reaction, the resulting reaction mixture was extracted with distilled water and ethyl acetate to yield the crude product. Finally, the crude product was further purified via column chromatography and recrystallisation to obtain compound **4** (yellow crystals, 1308 mg, 78.1%). ^1^H NMR (500 MHz, CDCl_3_, ppm) δ 3.77 (s, 6H), 6.79~6.81 (m, 4H), 7.01~7.03 (m, 4H), 7.15~7.17 (m, 1H), 7.41~7.44 (m, 1H), 7.57 (m, 1H), 7.62~7.69 (m, 2H), 7.71~7.73 (m, 1H), 8.03 (m, 1H), 8.15~8.17 (m, 1H), 8.90~8.97 (m, 2H); ^13^C NMR (125 MHz, CDCl_3_, ppm): δ 55.52, 111.42, 112.11, 114.67, 115.02, 122.89, 123.09, 123.78, 124.47, 124.90, 125.29, 129.72, 130.97, 131.96, 133.34, 140.89, 141.21, 142.03, 142.07, 144.63, 145.14, 145.18, 152.35, 155.24, 156.72.

Synthesis of ethyl 4-(8-(8-(bis(4-methoxyphenyl)amino)dibenzo[*b*,*d*]furan-2-yl)quinoxalin-5-yl)benzoate (**5**): The synthesis procedure of this compound referred to the synthesis process of compound **4**. Specifically, compound **4** (0.60 g, 1.00 mmol), (4-ethoxycarbonylphenyl)boronic acid (0.29 g, 1.50 mmol), Pd(PPh)_3_ (0.05 g, 0.04 mmol), K_2_CO_3_ (0.21 g, 1.50 mmol), tetrahydrofuran (16 mL), and H_2_O (2 mL) were combined. The above mixture was continuously heated and stirred at 70 °C overnight. Then, the crude product was subjected to extraction and column chromatography to gain compound **5** (yellow solid, 599 mg, 89.2%). ^1^H NMR (500 MHz, CDCl_3_, ppm) δ 1.42~1.45 (t, 3H), 3.79 (s, 6H), 4.41~4.46 (q, 2H), 6.80~6.82 (m, 4H), 7.03~7.05 (m, 4H), 7.16~7.18 (m, 1H), 7.44~7.45 (m, 1H), 7.60~7.61 (m, 1H), 7.67~7.68 (m, 1H), 7.75~7.77 (m, 1H), 7.78~7.80 (m, 2H), 7.89~7.94 (m, 2H), 8.11 (m, 1H), 8.20~8.22 (m, 2H), 8.88~8.90 (m, 2H); ^13^C NMR (125 MHz, CDCl_3_, ppm): δ 14.42, 55.52, 61.01, 111.38, 112.10, 114.67, 115.06, 122.97, 123.71, 124.43, 125.01, 125.29, 129.37, 129.70, 129.85, 130.23, 130.41, 130.68, 132.70, 139.30, 141.09, 141.19, 141.25, 142.09, 142.92, 144.42, 144.54, 152.35, 155.23, 156.67, 166.56.

Synthesis of 4-(8-(8-(bis(4-methoxyphenyl)amino)dibenzo[*b*,*d*]furan-2-yl)quinoxalin-5-yl)benzoic acid (**DQC**): At room temperature, compound **5** (0.67 g, 1.00 mmol) was initially dissolved in a mixed solvent of tetrahydrofuran (THF) and methanol (MeOH) (60 mL, *V*_THF_:*V*_MeOH_ = 5:1). KOH (0.56 g, 10.00 mmol) was then added to the solution, and the resulting mixture was heated to 80 °C and stirred overnight. After the reaction was completed, the solvent in the mixture was removed through evaporation. A large quantity of distilled water was then added to the residue, leading to the formation of a new suspension. Subsequently, the pH of the suspension was adjusted by adding dilute hydrochloric acid until a yellow-green crude product was precipitated. Finally, the crude product was purified by recrystallisation, giving compound **DQC** (yellow granular crystal, 517 mg, 80.3%). ^1^H NMR (500 MHz, CDCl_3_, ppm) δ 3.78 (s, 6H), 6.80~6.81 (m, 4H), 7.03~7.04 (m, 4H), 7.16~7.18 (m, 1H), 7.43~7.45 (m, 1H), 7.59~7.60 (m, 1H), 7.66~7.68 (m, 1H), 7.75~7.77 (m, 1H), 7.82~7.84 (m, 2H), 7.90~7.94 (m, 2H), 8.11 (m, 1H); 8.25~8.27 (m, 2H), 8.90~8.91 (m, 2H); ^13^C NMR (125 MHz, CDCl_3_, ppm): δ 55.53, 111.40, 112.11, 114.67, 115.06, 122.97, 123.74, 124.46, 125.01, 125.29, 128.48, 129.84, 130.02, 130.32, 130.44, 130.85, 132.64, 139.13, 141.04, 141.28, 141.37, 142.10, 143.84, 144.45, 144.61, 152.37, 155.23, 156.70, 170.81.

Synthesis of 5-(8-(8-(bis(4-methoxyphenyl)amino)dibenzo[*b*,*d*]furan-2-yl)quinoxalin-5-yl)thiophene-2-carboxylic acid (**DQC-T**): Into a 25 mL two-necked round-bottomed flask, **4** (0.30 g, 0.5 mmol), 5-carboxythiophene-2-boronic acid (0.26 g, 1.50 mmol), Pd(PPh)_3_ (0.09 g, 0.08 mmol), and Na_2_CO_3_ (0.11 g, 1.00 mmol) were added. Subsequently, under an N_2_ atmosphere, ultra-dry DMF (15 mL) was added to the above round-bottomed flask and the reaction was conducted in an oil bath at 150 °C for 4 h. After the reaction was over and returned to room temperature, the reaction mixture was extracted with a large amount of distilled water and ethyl acetate to obtain a crude product. Finally, the crude product was purified by column chromatography (DCM: MeOH = 10:1) to give the compound **DQC-T** (yellow-green powder, 131 mg, 40.2%). ^1^H NMR (500 MHz, CDCl_3_, ppm) δ 3.79 (s, 6H), 6.81~6.82 (m, 4H), 7.03~7.05 (m, 4H), 7.16~7.19 (m, 1H), 7.43~7.45 (m, 1H), 7.60~7.61 (m, 1H), 7.66~7.68 (m, 1H), 7.75~7.77 (m, 1H), 7.82~7.83 (m, 1H), 7.91~7.93 (m, 1H), 7.95~7.96 (m, 1H), 8.11~8.12 (m, 1H), 8.27~8.28 (m, 1H), 8.97~9.00 (m, 2H); ^13^C NMR (125 MHz, CDCl_3_, ppm): δ 55.52, 111.43, 112.11, 114.67, 115.07, 122.97, 123.77, 124.48, 124.96, 125.29, 127.10, 128.13, 129.82, 130.51, 131.33, 132.32, 134.18, 139.81, 142.09, 143.64, 144.61, 144.84, 146.41, 152.37, 155.23, 156.76, 166.16.

Synthesis of 8,8′-(thiophene-2,5-diylbis(quinoxaline-8,5-diyl))bis(*N*,*N*-bis(4-methoxyphenyl)dibenzo[*b*,*d*]furan-2-amine) (**DQ-T-QD**): The synthesis process of this compound referred to the synthesis process of **DQC-T**. The specific dosages of the raw materials were as follows: **4** (1.51 g, 2.50 mmol), 2,5-thiophene diboronate (0.09 g, 0.50 mmol), Pd(dppf)Cl_2_ (0.12 g, 0.10 mmol), K_2_CO_3_ (2M, 2 mL), and ultra-dry DMF (10 mL). The reaction time and temperature were set to 80 °C and 10 h, respectively. Finally, the resulting mixture was subject to a simple extraction (extraction agent: EtOAc and H_2_O) and column chromatography (Petroleum Ether: EtOAc = 5:1) to afford **DQ-T-QD** (orange powder, 299 mg, 53.1%). ^1^H NMR (500 MHz, CDCl_3_, ppm) δ 3.77 (s, 12H), 6.79~6.81 (m, 8H), 7.02~7.03 (m, 8H), 7.14~7.17 (m, 2H), 7.42~7.43 (m, 2H), 7.57~7.58 (m, 2H), 7.63~7.65 (m, 2H), 7.70~7.72 (m, 2H), 7.83~7.85 (m, 4H), 8.05~8.06 (m, 2H), 8.11~8.13 (m, 2H), 8.86~8.87 (m, 4H); ^13^C NMR (125 MHz, CDCl_3_, ppm): δ 55.51, 111.31, 112.07, 114.67, 115.08, 122.93, 123.67, 124.37, 125.04, 125.28, 128.93, 129.77, 129.83, 130.81, 132.77, 141.15, 141.17, 142.11, 143.35, 144.55, 144.69, 144.75, 152.35, 155.21, 156.61.

### 3.2. Device Fabrication

Pretreatment of ITO conductive substrates: PET/ITO substrates (2 cm × 2 cm) were etched with zinc powder and hydrochloric acid, and then ultrasonically cleaned in deionized water, ethanol, and isopropanol sequentially for 15 min. Before the preparation of the NiOx layer, the above mentioned substrates were adhered to a rigid glass substrate and dried at 75 °C for 20 min, and finally subjected to a UV–ozone treatment lasting 20 min.

Preparation of NiO_x_ layer: NiO_x_ nanocrystals were synthesized according to our previous work [40]. The previously prepared NiO_x_ nanocrystals were further prepared into an aqueous solution with a concentration of 20 mg mL^−1^. The NiO_x_ aqueous solution was subsequently filtered and the resulting filtrate was spin-coated onto a clean PET/ITO substrate at 2000 rmp for 30 s. Immediately thereafter, it was transferred to a heating table at 120 °C for an annealing treatment for 15 min. After the preparation of the layer, the substrate was placed in a nitrogen-filled glove box.

Preparation of hole transport layer: The designed and synthesized HTMs (**DQC-T** and **DQ-T-QD**, 0.4 mg) were accurately weighed and completely dissolved in DMSO (1 mL). The prepared solutions were spin-coated onto the NiO_x_ layer at 4000 rpm for 30 s and then annealed at 100 °C for 15 min.

Preparation of perovskite absorbent layer: The perovskite precursor was prepared by mixing CsI, FAI, MABr, PbBr_2_, and PbI_2_ in a mixed solvent (*V*_DMF_:*V*_DMSO_ = 4:1) and then stirring overnight in a nitrogen glove box. The perovskite precursor was further deposited onto PET/ITO/NiO_x_/HTM substrates by a two-step spin-coating process, including spin-coating at 1500 rpm for 10 s and at 6000 rpm for 25 s. During the last 5 s of the spin-coating process, 200 μL of anisole was added dropwise. Finally, the obtained substrate was quickly transferred to a heating table and thermally annealed at 100 °C for 30 min.

Preparation of electron transport layer: The precursor solution of PCBM was prepared by homogeneously dispersing it in chlorobenzene solution at a concentration of 20 mg mL^−1^. Subsequently, the PCBM precursor solution and 0.5 mg mL^−1^ BCP isopropanol solution were spun-coated on the substrate sequentially at 2000 rpm and 6000 rpm, both for 30 s.

Vapor deposition of Ag electrode: The top electrode was prepared by thermally evaporating a silver layer of about 100 nm on the above substrate under a vacuum of 2 × 10^−4^ Pa. Finally, an antireflection film was attached to the incident side of the fPSCs.

### 3.3. Measurement and Characterization

^1^H NMR and ^13^C NMR spectra were recorded on a Bruker 500 MHz spectrometer. Thermogravimetric analysis (TGA) and the differential scanning calorimetry (DSC) curve were measured employing an SDT 650+Discovery simultaneous thermal analyzer and TA DSC250 Instrument, respectively, under nitrogen atmosphere with a heating rate of 10 °C min^−1^. Ultraviolet–visible (UV–vis) absorption spectra were tested by a Cary 5000 UV-Vis-NIR spectrometer. Fluorescence emission spectra were carried out on a Hitachi F-7000 fluorescence spectrophotometer (Chiyoda, Japan). Cyclic voltammetry (CV) curves were detected on a Tatsuwa CHI6001 electrochemical workstation under argon atmosphere (Kanazawa, Japan). The test was performed using a three-electrode system with a scan rate of 50 mV s^−1^, where a platinum sheet was chosen as the counter electrode, a glass carbon electrode was chosen as the working electrode, and Ag/AgCl was chosen as the reference electrode. Additionally, 0.1 M tetrabutylammonium hexafluorophosphate dichloromethane solution and ferrocene (Fc/Fc+) were the electrolyte and external standard, respectively. Fourier transform infrared (FTIR) spectra were gained in the attenuated total reflection (ATR) mode on a Thermo Scientific Nicolet iS50 spectrometer (Waltham, MA, USA). The film morphology images of perovskite layers deposited on different substrates were characterized adopting a Zeiss Sigma 360 scanning electron microscope (SEM, Oberkochen, Germany). X-ray diffraction (XRD) measurements were completed on a Rigaku Ultima-IV (Japan) X-ray diffractometer (Tokyo, Japan). The steady-state photoluminescence (PL) spectra and time-resolved photoluminescence (TRPL) spectra were collected using a FLS1000 fluorescence spectrometer (Edinburgh instruments Ltd., Edinburgh, UK) with a 468 nm excitation wavelength. The current density–voltage (*J*–*V*) curves of the fPSCs were provided by a Keithley 2400 source meter under simulated AM 1.5 illumination (100 mW cm^−2^). The external quantum efficiency (EQE) spectra were conducted on a QE-R-3010 system.

### 3.4. Density Functional Theory (DFT) Calculations

The optimal molecular configurations, electron cloud distributions, and HOMO and LUMO energy levels of the HTMs were obtained based on DFT calculations. The computational procedures, methods, and benchmarks here were corresponded to Gaussian 09, B3LYP, and def2-SVP, respectively.

Theoretical calculations of the dipole moments of the molecules were performed at the B3LYP and def2-SVPD levels by means of the Gaussian 16 computational software with Grimme’s empirical D3BJ dispersion correction. In addition, the electrostatic potentials were further obtained by Multiwfn 3.8 (dev) calculations based on checkpoint files in Gaussian format and then plotted by VESTA 3.5.8.

## 4. Conclusions

To conclude, a triple design strategy was proposed to develop two quinoxaline-based HTMs (**DQC-T** and **DQ-T-QD**). The two HTMs were differentiated based on symmetry or asymmetry of structure and the presence or absence of an anchoring group (carboxyl group). Here, the integration of an S···N noncovalent conformation lock, D-A molecular skeleton, self-assembly, or conjugation engineering generated a synergistic effect that changed the HTMs’ properties and their device performance in a favorable direction. Thiophene as a π-bridge could form S···N noncovalent conformation locks with nitrogen atoms on adjacent quinoxaline units via its electron rich sulfur atoms, significantly reducing the corresponding dihedral angle to 3.51° for **DQC-T** or 0.47° for **DQ-T-QD**. Thereby, the molecular planarity was improved compared to **DQC**, which helped to achieve better interfacial contacts. The asymmetric D-A molecular skeleton (dibenzofuran-substituted diphenylamine-quinoxaline) endowed **DQC-T** with a markedly enhanced molecular dipole moment (*μ* = 5.80 D) and accelerated intramolecular charge transfer compared to the symmetric molecular skeleton. Moreover, the HTMs interacted with NiO_x_ and perovskite through electron-rich functional groups (e.g., carbonyl group, sulfur atom, and methoxy) in the structure, thereby inhibiting adverse interface reactions and obtaining high-quality perovskite films. As a result, the NiO_x_/**DQC-T**-based p-i-n fPSCs achieved a PCE of 18.12%, which exceeded that of the NiO_x_/**DQ-T-QD**-based device (PCE = 16.67%), NiO_x_/**DQC**-based device (16.75%), and NiO_x_-based device (PCE = 15.52%). In addition, the NiO_x_/**DQC-T**-based flexible device exhibited better environmental stability. This work successfully unveiled the structure–performance relationships involving noncovalent conformation locks and structural symmetry, and demonstrated that the basic properties of materials and device performance could be effectively improved by adopting an appropriate combination of molecular structure design strategies.

## Figures and Tables

**Table 1:** Photophysical, electrochemical, and thermal properties of HTMs.

HTMs	Experiment Data							Calculation Data ^e^	
	*λ*_max_ ^a^ (nm)	*λ*_em_ ^a^ (nm)	*E*_g_ ^b^ (eV)	*T*_d_ (℃)	*T*_g_ (℃)	HOMO ^c^ (eV)	LUMO ^d^ (eV)	HOMO (eV)	LUMO (eV)
**DQC-T**	303	488	2.77	243	-	−5.29	−2.52	−4.87	−2.72
**DQ-T-QD**	304	462	2.98	408	-	−5.31	−2.33	−4.76	−2.55

^a^ Measured in CHCl_2_ solution; ^b^ $E_{g}=1240/\lambda_{edge}$ (absorption edge of the materials in CHCl_2_ solution); ^c^ $E_{HOMO}=-|E_{ox}-E_{\left(Fc/{Fc}^{+}\right)ox}+5.11|$; ^d^ $E_{LUMO}=E_{HOMO}+E_{g}$; ^e^ Gaussian 09 at B3LYP and def2-SVP level.

**Table 2:** Photovoltaic performance parameters of devices based on different HTMs.

HTMs	*V*_oc_ (V)	*J*_sc_ (mA cm^−2^)	FF (%)	PCE_max_ (%)	*J*_sc-in_ (mA cm^−2^)
**NiO_x_**	1.05	22.36	65.96	15.52	21.10
**NiO_x_/DQC**	1.08	21.99	70.82	16.75	21.41
**NiO_x_/DQC-T**	1.09	22.28	74.44	18.12	22.31
**NiO_x_/DQ-T-QD**	1.04	23.25	69.10	16.67	22.85

## Data Availability

The original contributions presented in this study are included in the article/Supplementary Materials. Further inquiries can be directed to the corresponding author.

## Supplementary Materials

The following supporting information can be downloaded at: https://www.mdpi.com/article/10.3390/molecules30051129/s1, Figure S1: ^1^H NMR spectrum of **DQC-T**; Figure S2: ^13^C NMR spectrum of **DQC-T**; Figure S3: ^1^H NMR spectrum of **DQ-T-QD**; Figure S4: ^13^C NMR spectrum of **DQ-T-QD**; Figure S5: The electron distributions in HOMO and LUMO energy levels of **DQC-T** and **DQ-T-QD** from DFT calculation; Figure S6: The DSC curves; Figure S7: (a) The normalized UV-visible absorption spectrum; (b) raw UV-visible absorption spectrum in CHCl_2_; Figure S8: Cyclic voltammogram curves of **DQC-T** and **DQ-T-QD**; Figure S9: Fluorescence emission spectra of **DQC-T** and **DQ-T-QD**; Figure S10: SEM images of perovskite films deposited on different substrates (Magnification: 20k); Table S1: TRPL specifific parameters of perovskite films deposited on different substrates.
