# Supplementary material for: Triple Design Strategy for Quinoxaline-Based Hole Transport Materials in Flexible Perovskite Solar Cells
Source: Molecules. 2025 Feb 28;30(5):1129. doi: 10.3390/molecules30051129 (PMC11901842; doi:10.3390/molecules30051129)
Supplement: Supplementary file 1 [file molecules-30-01129-s001.zip › molecules-3488121-supplementary.pdf]

Supplementary Materials

Triple Design Strategy for Quinoxaline-Based Hole Transport Materials in Flexible Perovskite Solar Cells

Yuanqiong Lin [1], Zeyuan Gao [2], Xiaoshang Zhong [3], Yinghua Lu [3], Song Tu [3,*] and Xin Li [2,*]

[1] Pen-Tung Sah Institute of Micro-Nano Science and Technology, Xiamen University, Xiamen 361005, China; yuanqionglin@stu.xmu.edu.cn
[2] School of Electronic Science and Engineering, Xiamen University, Xiamen 361005, China; 36120231150453@stu.xmu.edu.cn
[3] College of Chemistry and Chemical Engineering, Xiamen University, Xiamen 361005, China; 36520231152088@stu.xmu.edu.cn (X.Z.); ylu@xmu.edu.cn (Y.L.)
* Correspondence: tusong@xmu.edu.cn (S.T.); lixin01@xmu.edu.cn (X.L.)

1. Structural characterization of HTMs

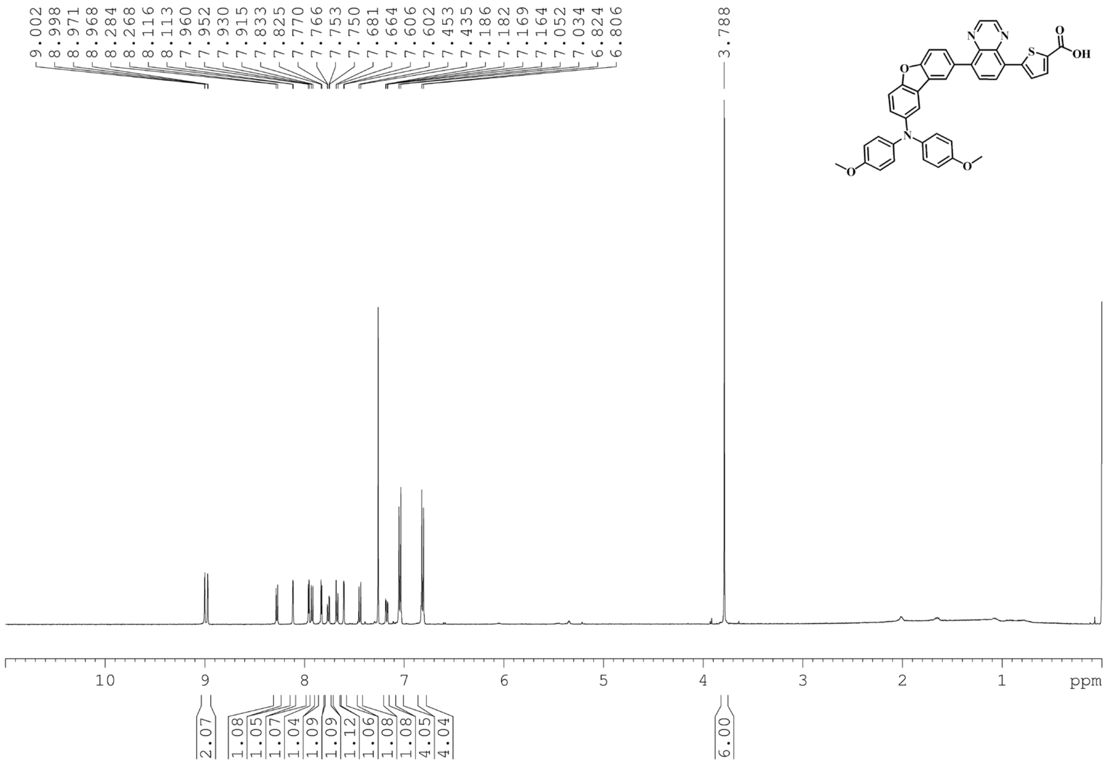

Figure S1. ^{1}H NMR spectrum of **DQC-T**.

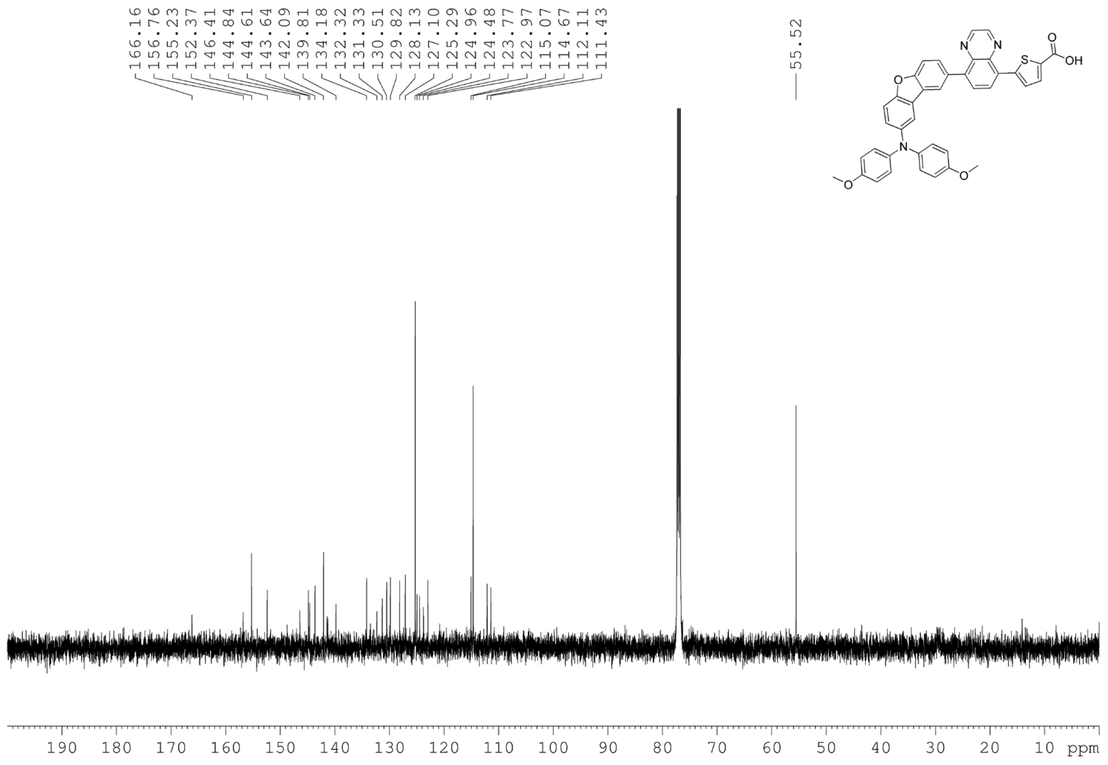

Figure S2 ^{13}C NMR spectrum of **DQC-T**.

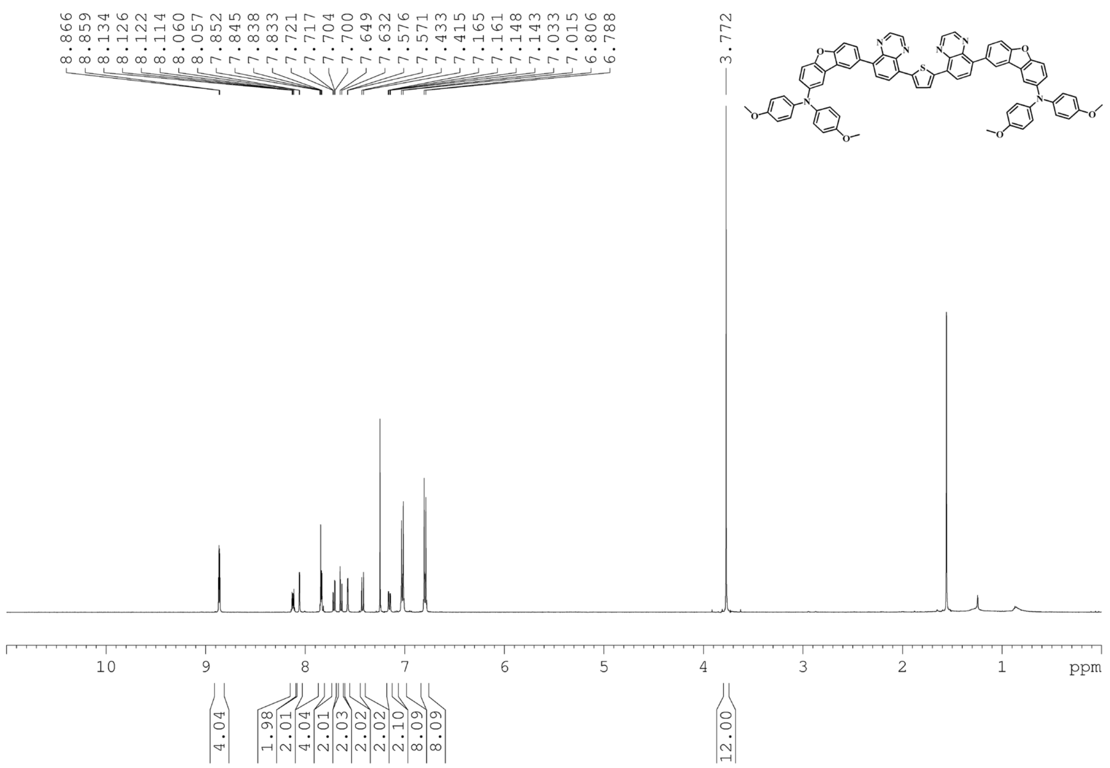

Figure S3. ^{1}H NMR spectrum of **DQ-T-QD**.

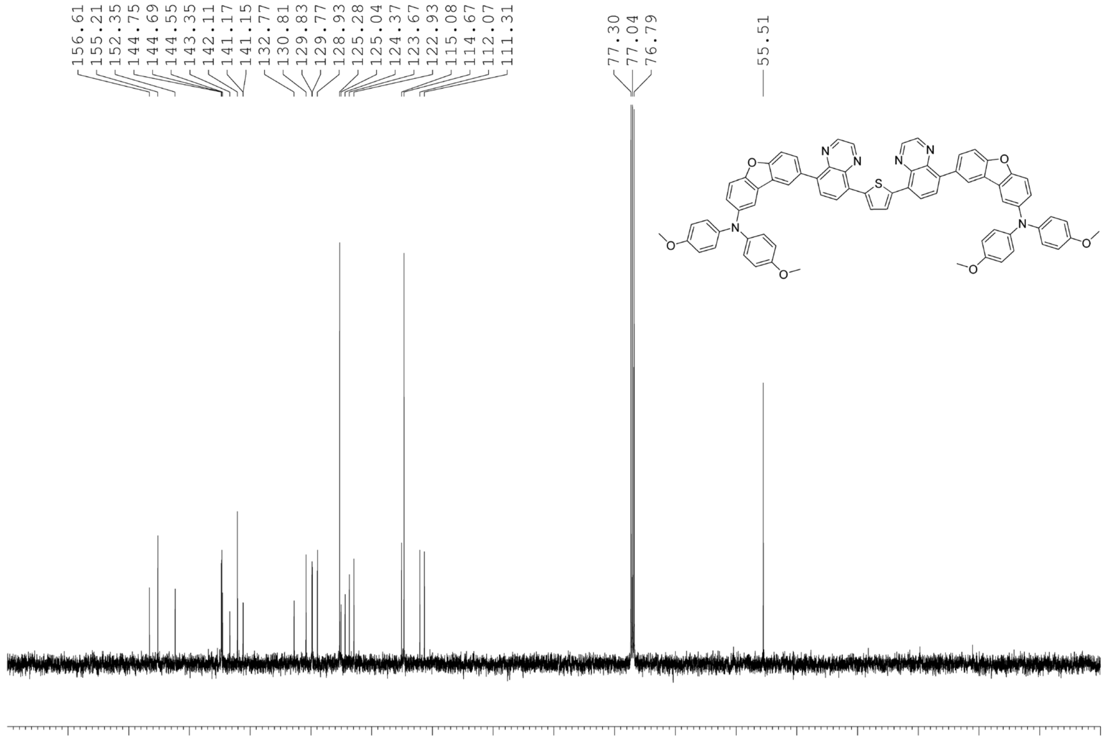

FigureS4. ^{13}C NMR spectrum of **DQ-T-QD**.

2. DFT calculation

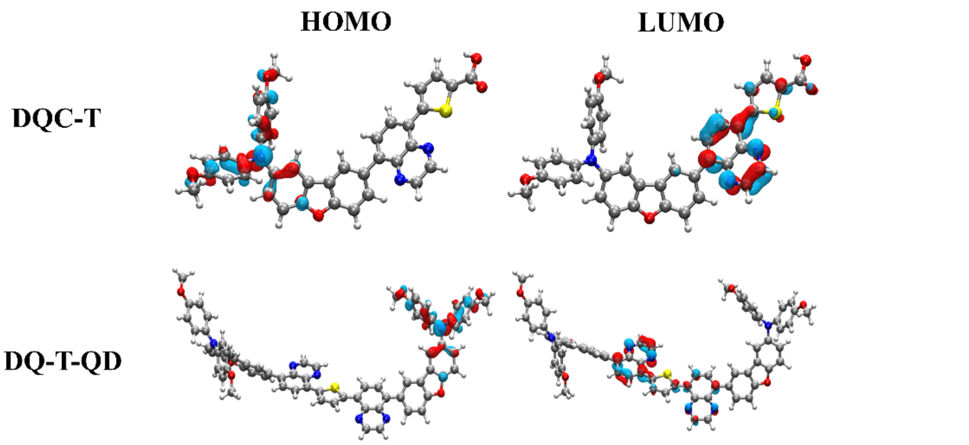

Figure S5. The electron distributions in HOMO and LUMO energy levels of **DQC-T** and **DQ-T-QD** from DFT calculation.

3. DSC curves

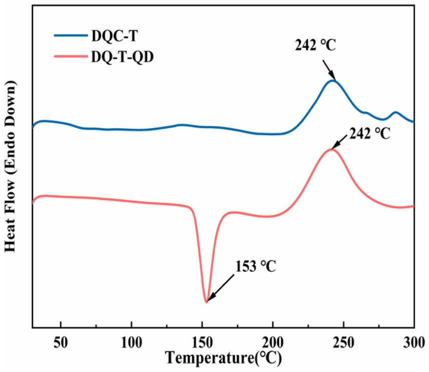

Figure S6. The DSC curves.

4. UV-visible absorption

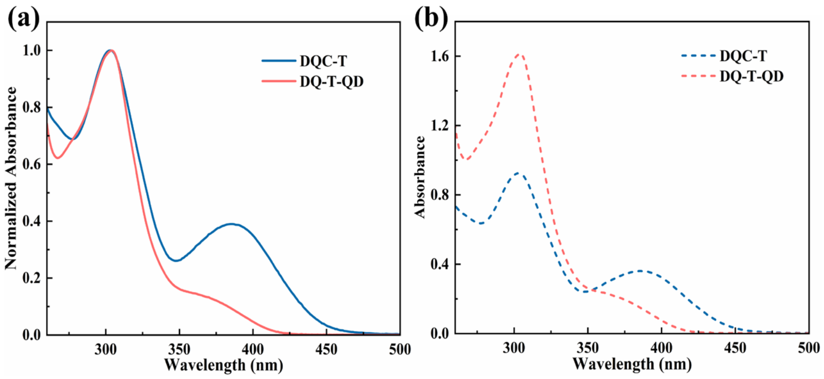

Figure S7. (a) The normalized UV-visible absorption spectrum; (b) raw UV-visible absorption spectrum in $CHCl_2$

5. Cyclic voltammogram

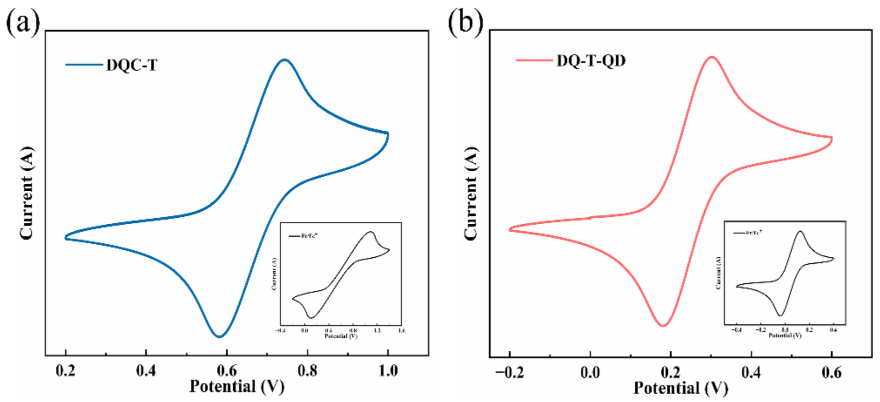

Figure S8. Cyclic voltammogram curves of **DQC-T** and **DQ-T-QD**.

6. Fluorescence emission spectra

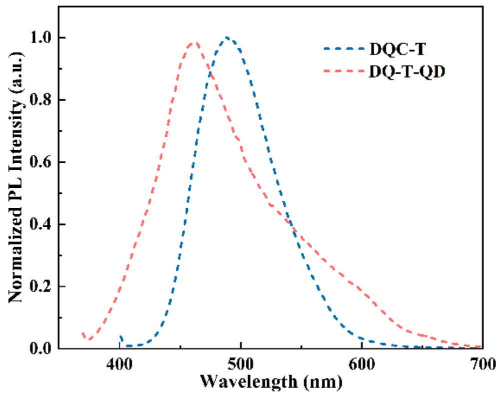

Figure S9. Fluorescence emission spectra of **DQC-T** and **DQ-T-QD**.

6. Perovskite film morphology

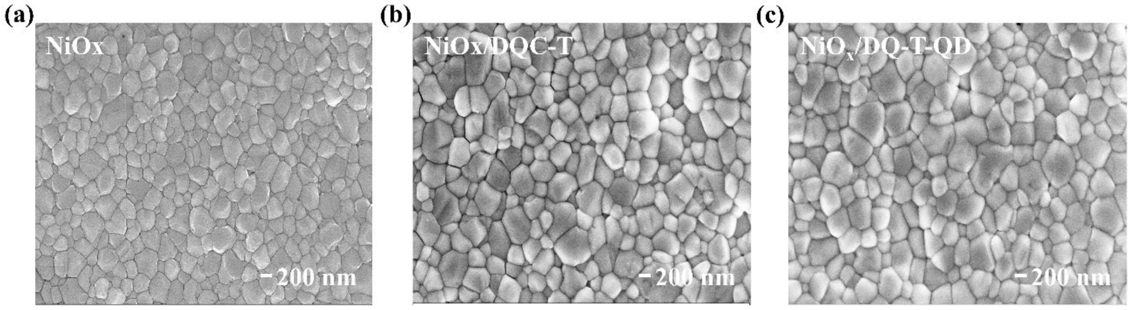

Figure S10. SEM images of perovskite films deposited on different substrates (Magnification: 20k).

8. TRPL fitting results

Table S1. TRPL specifific parameters of perovskite films deposited on different substrates.

Samples	A_1	τ_1 (ns)	A_2	τ_2 (ns)	τ_{ave}[a] (ns)
**NiO_x/perovskite**	0.99	332.07	0.24	3391.93	2503.48
**NiO_x/DQC/perovskite**	0.76	236.08	0.22	2146.09	1614.88
**NiO_x/DQC-T/perovskite**	1.19	147.21	0.27	1158.11	796.18
**NiO_x/DQ-T-QD/perovskite**	0.96	191.41	0.25	1262.62	865.68

[a] τ_{ave} meant the carrier average lifetime, derived from the following equation: $\tau_{ave} = (A_1\tau_1^2 + A_2\tau_2^2)/(A_1\tau_1 + A_2\tau_2)$
